# Analysing and meta-analysing time-series data of microbial growth and gene expression from plate readers

**DOI:** 10.1371/journal.pcbi.1010138

**Published:** 2022-05-26

**Authors:** Luis Fernando Montaño-Gutierrez, Nahuel Manzanaro Moreno, Iseabail L. Farquhar, Yu Huo, Lucia Bandiera, Peter S. Swain

**Affiliations:** 1 School of Biological Sciences, University of Edinburgh, Edinburgh, United Kingdom; 2 School of Engineering, University of Edinburgh, Edinburgh, United Kingdom; bioinformatics, GERMANY

## Abstract

Responding to change is a fundamental property of life, making time-series data invaluable in biology. For microbes, plate readers are a popular, convenient means to measure growth and also gene expression using fluorescent reporters. Nevertheless, the difficulties of analysing the resulting data can be a bottleneck, particularly when combining measurements from different wells and plates. Here we present omniplate, a Python module that corrects and normalises plate-reader data, estimates growth rates and fluorescence per cell as functions of time, calculates errors, exports in different formats, and enables meta-analysis of multiple plates. The software corrects for autofluorescence, the optical density’s non-linear dependence on the number of cells, and the effects of the media. We use omniplate to measure the Monod relationship for the growth of budding yeast in raffinose, showing that raffinose is a convenient carbon source for controlling growth rates. Using fluorescent tagging, we study yeast’s glucose transport. Our results are consistent with the regulation of the hexose transporter (HXT) genes being approximately bipartite: the medium and high affinity transporters are predominately regulated by both the high affinity glucose sensor Snf3 and the kinase complex SNF1 via the repressors Mth1, Mig1, and Mig2; the low affinity transporters are predominately regulated by the low affinity sensor Rgt2 via the co-repressor Std1. We thus demonstrate that omniplate is a powerful tool for exploiting the advantages offered by time-series data in revealing biological regulation.

## Author summary

Time series of growth and of gene expression via fluorescent reporters are rich ways to characterise the behaviours of cells. With plate readers, it is straightforward to measure 96 independent time series in a single experiment, with readings taken every 10 minutes and each time series lasting tens of hours. Analysing such data can become challenging, particularly if multiple plate-reader experiments are required to characterise a phenomenon, which then should be analysed simultaneously. Taking advantage of existing packages in Python, we have written code that automates this analysis but yet still allows users to develop custom routines. Our omniplate software corrects both measurements of optical density to become linear in the number of cells and measurements of fluorescence for autofluorescence. It estimates growth rates and fluorescence per cell as continuous functions of time and enables tens of plate-reader experiments to be analysed together. Data can be exported in text files in a format immediately suitable for public repositories. Plate readers are a convenient way to study cells; omniplate provides an equally convenient yet powerful way to analyse the resulting data.

## Introduction

Microbes live in dynamic environments [1], and time-series data is invaluable for measuring and understanding their response to change [2]. Such extracellular changes may be applied by the experimenter or brought about by the microbes themselves as they grow and consume extracellular resources. A common, medium throughput way to phenotype microbial responses over time is to use plate readers, which continually measure both optical density and fluorescence from wells in microplates.

Although using growth curves to evaluate microbial behaviour is long established [3], researchers now characterise the response mechanistically through combining such data with tagging by fluorescent proteins. These markers are used either to monitor the proteins of a system of interest or to follow the activity of exogenous copies of their promoters. With plate readers, the fluorescence per cell as a function of time can then be determined in a range of extracellular conditions. Such data are rich enough to reveal, for example, the structure of a biochemical network [4, 5], to discriminate specific from generic responses depending on growth rate [6–8], to investigate regulatory conflicts generated by applying a pair of antibiotics [9], and to demonstrate that expression of metabolic genes is controlled by only a few metabolites [10].

Nevertheless, averaging over different wells, estimating growth rates as a function of time, correcting for autofluorescence, determining the fluorescence per cell, and particularly combining data from multiple experiments can all become bottlenecks because of the amount of data generated. For example, with measurements taken every 10 minutes, experiments lasting say 24 hours, and 96 wells, one plate-reader experiment will generate almost 14,000 data points, and multiple experiments are typically required to characterise a biological system.

Here we present software that performs a comprehensive analysis of plate-reader data via established methods and data structures using the free programming language Python. We illustrate the power of our approach both by finding the Monod curve for budding yeast growing in the sugar raffinose and by studying glucose transport in yeast, where cells need at least one of seven transporters to grow aerobically on glucose [11]. Using Green Fluorescent Protein (GFP) to tag genes HXT1–7 and using mutants of the regulatory network, we show that with times-series data we are able not only to recover the regulatory control known from biochemical experiments, but also to clarify previously ambiguous regulation.

## Design and implementation

### The omniplate software

The omniplate Python module takes as input the data file produced by a plate reader, such as those made by Tecan, and a ‘contents’ file, which is a Microsoft Excel file with a 8 × 12 table that gives the strains and extracellular media in each well in a 96-well plate. Using the Python module pandas, omniplate creates three data frames, each a two dimensional, labelled data structure similar to a spreadsheet or to a table in SQL. The first data frame contains the raw time-series data, the second contains processed time-series data, and the third contains summary statistics calculated from the processed data, such as maximal growth rates and lag times.

The user has great flexibility to customise analysis because omniplate is built on the core packages both of scientific Python—numpy, scipy, and matplotlib—and of Python’s tools for data science—pandas and seaborn. To ensure reproducibility, omniplate creates a log of the methods called by a particular instance, which is exported as plain text. Further, it allows all figures to be saved into a single PDF file.

With omniplate, the user can automatically perform multiple tasks:

#### Ignore any corrupted wells

**Fig 1:**
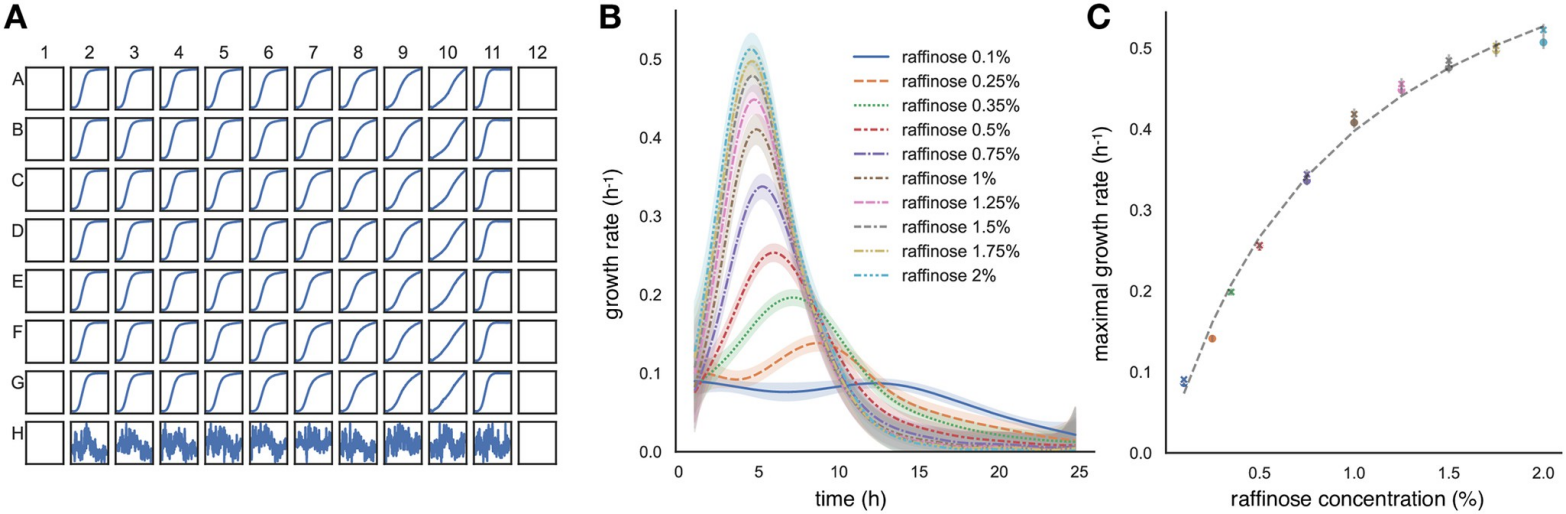
Using omniplate, it is straightforward to simultaneously analyse data from multiple wells in the same plate and from multiple plates. **A** An overview of the measurements of OD in each well of an experiment investigating growth of budding yeast in the sugar raffinose. Wells at the edge of the plate have been left empty because the media in these wells can evaporate more than others, at least for our plate reader. The OD measured in each well is plotted as a function of time. We used the wells in the last row for normalisation. They contain only media and have a different scale on the *y*-axis. **B** omniplate estimates growth rate as a function of time using a Gaussian process [17]. To determine yeast’s growth rate in different concentrations of raffinose, data from two plates with seven replicates for each raffinose concentration per plate was combined. Each concentration is present in both plates except for 0.25% and 0.35% raffinose. We show the average for the two plates of the mean growth rate inferred from all the relevant wells in each plate. Data from the first hour of the experiments was not used to estimate growth rates because of fluctuations in the measured OD. Shaded errors are standard deviations. **C** Yeast’s growth in raffinose obeys Monod’s equation (dashed line) with a best-fit *K*_*S*_ of 1.0% (20 mM); the best-fit corresponding growth-rate parameter is 0.82 h^−1^. The local maximal growth rate for each concentration for each plate is shown.

Data in some wells of the plate can be corrupted, perhaps by being contaminated or because of condensation. Using plotplate, which generates a plot of either the OD or fluorescence of all the wells in a 8 × 12 format (Fig 1A), the user can identify any such wells by eye and remove them from the analysis using ignorewells.

#### Correct OD to be proportional to numbers of cells

For the optical density (OD) to be proportional to the number of cells—the Beer-Lambert law—light passed through the cells should only be absorbed, but most microbes scatter light [12]. This scattered light is typically captured by the detectors in the plate reader when there are few cells in each well. As the cell populations become more dense, however, light is scattered multiple times, and all of it is no longer detected, causing the Beer-Lambert law to fail [12].

One way to correct for this failure is to calibrate [12, 13]. A dense culture of cells can be diluted in a series, samples measured in the plate reader, and the measured OD compared to the initial culture’s OD multiplied by the appropriate dilution factor. Any discrepancies are generated by a non-linear relationship between the OD and the number of cells. By plotting the measured OD on the *x*-axis and the expected OD on the *y*-axis, we generate a calibration curve, which we fit using a Gaussian process to interpolate to ODs that have not been measured [14].

We use this calibration curve to correct all measured ODs, but the units of the corrected OD are determined by the OD of the initial dense culture we used to generate the dilution series. Rather than keep this arbitrary value, we rescale so that the measured OD and correct OD are the same at an OD of 0.3. This rescaling means that the corrected OD varies linearly with cell numbers for sufficiently small ODs, as it should [12]. The value of 0.3 is also arbitrary, but recovering the linear scaling at sufficiently low ODs ensures that the units are such that the corrected OD is larger than the measured OD, as expected intuitively.

With omniplate, correctOD fits a dilution series and corrects all measured ODs. We include data for a dilution series for haploid budding yeast in glucose, but the user can provide another via a text file comprising two columns: one of the measured ODs and the other of the corresponding dilution factors.

#### Correct for the OD and fluorescence of the media

The OD and fluorescence of wells containing medium only are subtracted from the OD and fluorescence of wells containing medium and microbes by correctmedia. We use a Savitzky-Golay filter to smooth measurements as a function of time.

#### Correct for autofluorescence

Two methods correct for autofluorescence, one specialised to Green Fluorescent Protein (GFP) and one more general, if potentially less accurate. Both require multiple technical replicates (the same strain in the same conditions in more than one well) and wells with strains not carrying any fluorescent markers. To unambiguously detect any outliers, we use five wells for the tagged strain. For the untagged strain, we use seven wells because data from this strain corrects all fluorescent strains and must be reliable. Both methods are called through correctauto.

When measuring GFP, we perform the correction with linear unmixing, which estimates the autofluorescence using measurements from the GFP-tagged strains themselves [15]. We excite at a single wavelength, but measure emissions both at the wavelength appropriate to GFP and at a higher wavelength where emissions mostly come from autofluorescence. Data from the untagged strain indicates how autofluorescence at the higher wavelength is related to autofluorescence at GFP’s wavelength. Using this relationship and the measured fluorescence of the tagged strains at the higher wavelength, we correct for autofluorescence [15]. Where tested, this method performs better than alternatives, particularly when fluorescence is low [16].

In practice, there is some GFP emission at the higher wavelength, but the ratio of GFP emissions at the two wavelengths can be measured and included in the correction [15]. To combine measurements from multiple replicates, we use a Savitzky-Golay filter to smooth *r*_*a*_, the ratio of emissions at the two wavelengths for the untagged strain, as a function of OD. We interpolate the value of this ratio to the OD of tagged strains at each time point and so perform the correction via [15]

$$\begin{array}{c} f_{\text{corr}}=\frac{r_{a}f_{525}-f_{585}}{r_{a}-r_{g}} \end{array}$$ (1)

where *r*_*g*_ is the measured ratio of GFP’s emission at 585 nm to that at 525 nm. Here *f*_525_ is the emission of a tagged strain at the wavelength for GFP and *f*_585_ is its emission at the higher wavelength of 585 nm. The error at each time point is estimated as the variance of the corrected fluorescence of the tagged strain over its replicates.

We estimate the fluorescence per cell by dividing the corrected fluorescence for each replicate by that replicate’s OD and taking the mean over the replicates. The error is given by the corresponding variance.

As an alternative and for other fluorophores, such as mCherry, we estimate autofluorescence as the fluorescence of the untagged strain as a function of its OD. To perform the correction, we subtract this autofluorescence interpolated to the OD of a tagged strain from the tagged strain’s fluorescence at each time point [8]. We use a Savitzky-Golay filter to smooth the fluorescence of the untagged strain’s replicates and estimate errors by the variance of the corrected fluorescence of the tagged strain over its replicates.

Both methods check consistency by returning the corrected fluorescence of the untagged strain, which should fluctuate around zero.

#### Estimate growth rates

To estimate growth rates using data from multiple replicate wells, we use a Gaussian process, which makes only weak assumptions on the mathematical form of the growth curve and propagates the errors in measuring the OD to the errors in the inferred growth rates [17]. The getstats method not only determines the specific growth rate as a function of time, but also its time derivative and calculates statistics, such as the maximal growth rate, the local maximal growth rate (at a true maximum and not at the beginning or end of the experiment), and the lag time, as well as their errors, which are estimated using bootstrapping. We use a Matern covariance function [14], which only constrains the growth curve to be twice differentiable—by growth curve we mean the logarithm of the OD because we estimate the specific growth rate. The user can select a covariance function that is a squared exponential or neural-network-like [14] if preferred.

The getstats method can be applied to other data to estimate its time derivative, such as the fluorescence per OD.

#### Average over experiments

Either data from multiple experiments can be imported and processed simultaneously or previously processed data can be loaded at once. Data can be exported to text and JSON files or to Microsoft Excel spreadsheets.

Even if the plate reader is programmed identically, different experiments with the same strain in the same condition typically have measurements at slightly different time points. Therefore to average over experiments, using addcommonvar, we determine a common time variable and interpolate measurements not made at this common time to the common time. Averaging is then performed for each common time point.

#### Create new columns in the data frames

To enable plotting, it is often useful to add columns to the data frames specifying the values of variables that the experimenter has systematically changed, such as the concentration of a nutrient or antibiotic. These numerical values can be automatically extracted using addnumericcolumn.

### Experimental methods

Although strains were kept in plates of XY media with 2% glucose, we prepared pre-cultures by inoculating single yeast colonies in SC medium (Table A in S1 Text), complemented with 2% pyruvate—cells then respire and perform gluconeogenesis, and so we avoid any glucose-dependent effects. We incubated such pyruvate cultures for 24 hours, then diluted and grew cells in fresh medium for another 21 hours, and then again diluting and growing in fresh medium for 3–5 hours to reactivate growth.

We measured optical density in either a Tecan F200 or M200 plate reader in 96-well microplates (Thermo-Fisher) with 200 *μ*l of cell culture using low fluorescence SC medium (Table A in S1 Text). Individual OD measurements are close to identical between the machines, and we measured fluorescence exclusively in a M200 machine.

All strains were derived from BY4741 (Table B in S1 Text).

## Results

### Characterising the growth of budding yeast in raffinose

To illustrate omniplate, we measure the Monod curve for budding yeast growing with raffinose as the carbon source. Monod observed that the mid-log growth rate of *Escherichia coli* often obeys a hyperbolic relationship with the concentration of a carbon source [3]. Writing *μ* as the mid-log growth rate, he observed that

$$\begin{array}{c} \mu =\frac{\mu_{\text{max}}S}{K_{S}+S} \end{array}$$ (2)

where *S* is the concentration of the carbon source, *μ*_max_ is the maximal growth rate, and *K*_*S*_ is a constant characterising the carbon source. Eq 2 shows that growth increases with the availability of carbon until carbon is no longer limiting.

Many researchers are interested in determining how a phenomenon of interest is affected by growth rate [18], and the Monod constant, *K*_*S*_, is useful because it indicates the concentration of the carbon source around which a change in concentration will modify growth rate. For budding yeast, for example, the *K*_*S*_ for glucose is around 0.15 mM or 0.003% [19], making growing in glucose a poor choice for studying the effect of growth rate because such small concentrations are difficult to prepare.

As an alternative, we used raffinose, a trisaccharide for which only the fructose moiety is metabolised [20], and performed two plate-reader experiments (Fig 1A) where the OD was measured for cells growing in 10 different raffinose concentrations over 24 hours. Using omniplate, we worked on the two datasets simultaneously, correcting both for the OD of the media and for non-linearities between the OD and cell numbers and then estimating the growth rate using data from wells containing technical replicates (Fig 1B). By plotting the local maximal growth rate—the growth rate at a time where the growth rate has a true peak—versus the initial concentration of raffinose for the corresponding wells, we see that the results are well described by a Monod curve (Fig 1C) with a *K*_*S*_ of 20 mM or 1%, a more manageable concentration than for glucose.

### A time-series analysis of budding yeast’s response to glucose

To illustrate further omniplate’s capabilities, we performed a fluorescence-based study of one aspect of yeast’s response to glucose. Yeast chiefly uses seven hexose transporters (Hxts) to grow on glucose [21, 22]—so many possibly to mitigate rate-affinity tradeoffs [23], and we created strains where one of those transporters is tagged with GFP.

**Fig 2:**
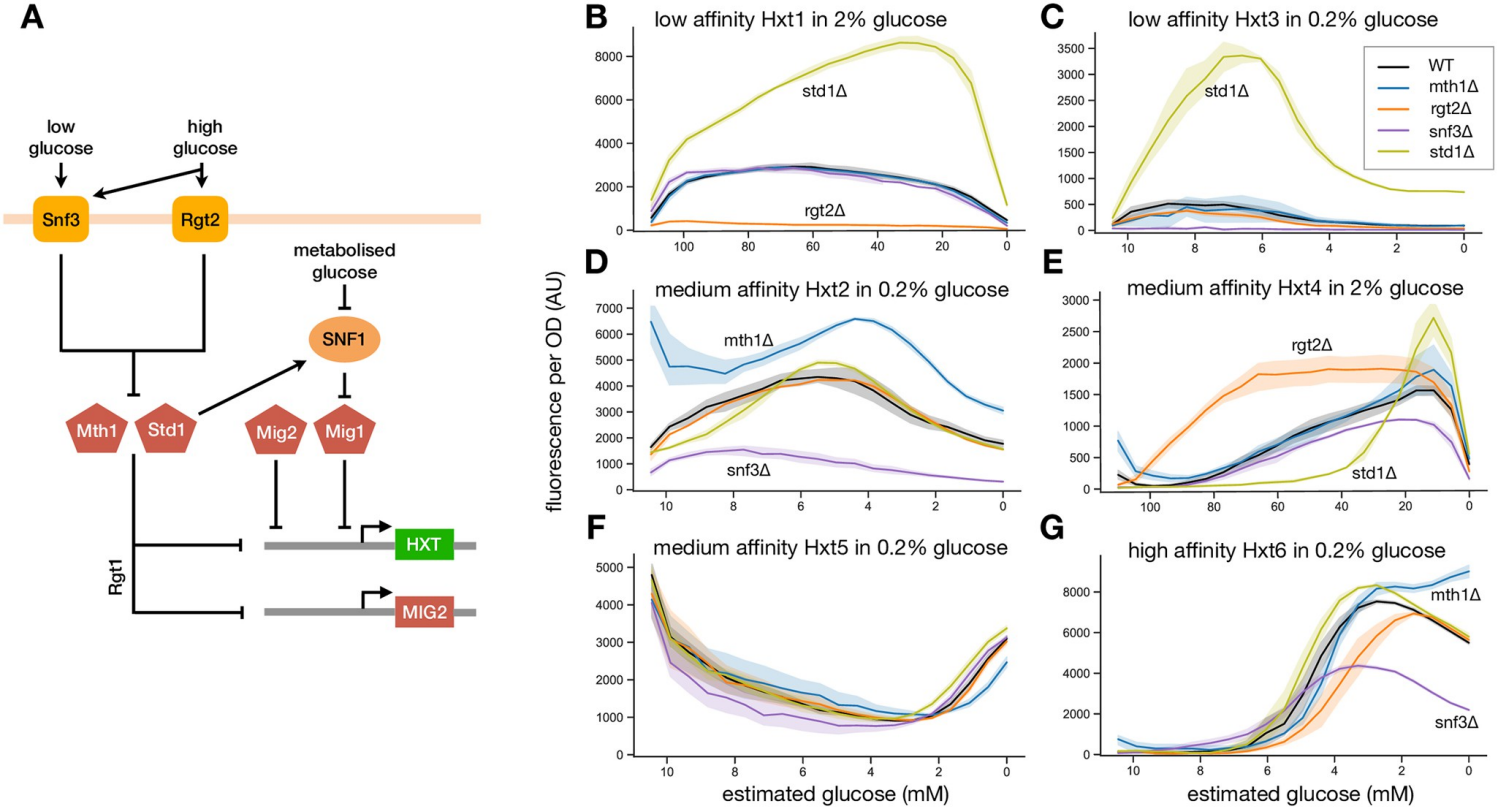
Measuring over time GFP-tagged outputs of a system of interest both in wild-type and in mutant strains missing components of the regulatory network can reveal much of the network’s structure. We illustrate with the network that controls the expression of hexose transporters in budding yeast. **A** Expression of the HXT genes is controlled by at least two subsystems, which together regulate the activities of four repressors. Extracellular glucose is sensed by the Snf3-Rgt2 network: Snf3 is a low affinity sensor; Rgt2 has a high affinity. Mth1 and Std1 are co-repressors that interact with the transcription factor Rgt1 to repress the HXTs. Extracellular glucose is sensed by the Snf3-Rgt2 system, which inactivates Mth1 and Std1 and increases HXT expression. Via Rgt1, the system also regulates MIG2, which encodes another repressor. Intracellular glucose is sensed by the SNF1 kinase complex, which when active phosphorylates a final repressor, Mig1. When phosphorylated, Mig1 exits the nucleus and cannot repress the HXTs. **B-G** Using plate readers we followed levels of Hxts tagged with GFP. We transformed the resulting time-series measurements first into GFP per cell and then, approximately, into functions of extracellular glucose by estimating glucose from the culture’s OD. Time increases from left to right as glucose falls and the OD increases. At the extreme left—corresponding to *t* = 0, the level of each Hxt is the level attained in the pyruvate used for pre-growth. Data are the average of at least two experiments, each with four biological replicates. Errors are 95% confidence intervals. **B** HXT1 is strongly repressed by Std1, and Rgt2 is required to relieve Std1’s repression. **C** HXT3 behaves similarly to HXT1, but expresses in low glucose and requires Snf3 to do so. **D** HXT2 is strongly repressed by Mth1 but not Std1, and Snf3 is required to relieve Mth1’s repression. **E** HXT4 is repressed by Mig1 and Mig2 in high glucose because its levels both decrease when STD1 is deleted and increase when RGT2 is deleted. **F** HXT5 is at best weakly regulated by the network—all mutants behave similarly to the wild-type strain. **G** HXT6 is strongly repressed by Mth1 but not Std1 in low glucose. HXT7, which also encodes a high affinity transporter, behaves similarly (Fig G in S1 Text).

Although the transporters are known to have different affinities for glucose [24, 25], the control of their expression is only partly understood. Expression is predominately regulated by two subsystems (Fig 2A) [22].

The first is the Snf3-Rgt2 subsystem, comprising a low affinity sensor for glucose—Rgt2, a high affinity sensor—Snf3, and the transcriptional regulator Rgt1, together with its two co-repressors Mth1 and Std1 [26]. If Rgt1 forms a complex at a HXT promoter with at least one of Mth1 and Std1 [27], it inhibits expression [28, 29]. Sufficient extracellular glucose inactivates the co-repressors Mth1 and Std1 via the Rgt2 and Snf3 sensors [30]. Mth1 is inactivated by being degraded [31]; Std1 is inactivated not only by being degraded [32] but also by condensing into cytoplasmic granules [33]. With Mth1 and Std1 inactivated, the HXT genes express.

The Snf3-Rgt2 subsystem negatively regulates another repressor gene MIG2 [34], which therefore expresses in glucose. Its protein product also inhibits HXT expression.

The second subsystem is SNF1 kinase, a complex of three proteins known as AMP kinase in higher eukaryotes. SNF1 responds to both low intracellular glucose [21] and to active Std1 [33, 35] (Fig 2A). It promotes HXT expression by phosphorylating the repressor Mig1, preventing its binding to the HXT promoters [34, 36].

We sought to understand how these four repressors—Std1, Mth1, Mig1, and Mig2—differentially regulate the HXTs, whose expression peaks at different glucose concentrations [28, 37–41]. Using GFP-tagging, we followed the Hxt levels in the wild-type strain and in strains where either one of the sensors Snf3 or Rgt2 or one of the co-repressors Mth1 or Std1 was deleted. To set an initial, basal level of glucose transport, each strain was pre-cultured in pyruvate where cells do not use glycolysis. We performed nine different experiments and analysed the data from all nine plates together with omniplate.

Although the HXTs respond to extracellular glucose, we cannot straightforwardly measure its concentration in this many experiments, and instead we estimated the concentration from each culture’s OD. Assuming that a fixed amount of glucose must be imported for a cell to replicate, we estimated the glucose concentration as a linear decreasing function of the OD [42]:

$$\begin{array}{c} g\left(t\right)=g_{0}\cdot \frac{OD_{\text{max}}-OD\left(t\right)}{OD_{\text{max}}-OD_{\text{min}}}. \end{array}$$ (3)

This function has the culture’s initial glucose concentration, *g*_0_, at the minimal OD when the experiment begins and is zero when the experiment finishes because the OD has then plateaued. Although the estimate is crude, plotting the fluorescence per cell as a function of the estimated glucose concentration emphasises how the deleted genes affect the transporters’ levels (Fig 2B–2G).

Building on previous work [22, 26, 28], these time-series data allow us to draw three conclusions about how the glucose sensors Rgt2 and Snf3 regulate the two co-repressors Mth1 and Std1:

1. The low affinity sensor Rgt2 predominately inactivates the co-repressor Std1. Deleting the gene for STD1—but not the gene for the other co-repressor MTH1—substantially increases the levels of Hxt1 and Hxt3 (Fig 2B and 2C), implying that HXT1 and HXT3 are repressed almost entirely by Std1 in these concentrations of glucose. Rgt2 likely inactivates Std1 because HXT1’s expression in 2% glucose is only completely inhibited when RGT2 is deleted (Fig 2B). Nevertheless, the high affinity sensor Snf3 also inactivates Std1, if weakly in 0.2% glucose because HXT3’s expression in 0.2% glucose is only completely inhibited when SNF3 is deleted (Fig 2C).
2. Mth1 is inactivated at lower concentrations of glucose than Std1. Hxt1 and Hxt3 are absent in pyruvate in the *mth1*Δ and *std1*Δ strains (data on extreme left of Fig 2B and 2C), and so each is repressed by both Mth1 and Std1. Yet in 0.2% glucose, Hxt3 levels increase if STD1 but not if MTH1 is deleted (Fig 2C). The difference is consistent with Mth1, but not Std1, being sufficiently inactivated to no longer repress at this glucose concentration. In agreement, the MTH1 gene is known to be repressed in glucose whereas STD1 is not [43].
3. The high affinity sensor Snf3 predominately inactivates Mth1. Focusing on Hxt2 (Fig 2D), HXT2 is strongly regulated by Mth1 because only deleting MTH1 increases its levels in pyruvate (data on extreme left). Considering the deletions for the two sensors, removing SNF3 causes a weaker response than the wild-type, and so Snf3 must inactivate Mth1. There is, however, still some response in the *snf3*Δ strain (Fig 2D), and so Rgt2 does inactivate Mth1, if weakly.

Although perhaps not so explicitly stated before, these results dovetail with the general understanding of the Snf3-Rgt2 network available from the literature.

We can also infer regulation by the other two repressors Mig1 and Mig2 from our measurements. Perhaps counterintuitively, deleting the gene for the co-repressor STD1 can actually decrease an Hxt’s levels compared to the wild-type, and if so deleting the sensor RGT2 increases its levels. This phenomenon is most obvious for Hxt4 (Fig 2E), but occurs too for Hxt2, Hxt6, and Hxt7 (Figs B, F, and G in S1 Text).

Two mechanistic explanations both imply that the HXT is regulated by Mig1 and Mig2. Std1 inhibits repression by Mig1 and Mig2 both by promoting SNF1’s activity and by repressing the MIG2 gene (Fig 2A). Deleting STD1 therefore reduces an Hxt’s levels if that Hxt’s promoter is repressed by Mig1 and Mig2. In contrast, deleting RGT2 hyper-activates Std1, increasing its inhibition of Mig1 and Mig2 and raising the Hxt’s levels. Lower levels in the *std1*Δ strain with higher levels in the *rgt2*Δ strain therefore imply Mig1 and Mig2 regulate a HXT, and the absence of this effect implies they do not.

Our data therefore give new insight into which of the four repressors regulate each HXT gene:

1. The genes for the low affinity transporters HXT1 and HXT3 are repressed by Std1 and also by Mth1 in sufficiently low glucose, but only weakly if at all by Mig1 and Mig2 (Fig 2B and 2C, Figs A and C in S1 Text).
2. HXT4 is repressed by Mth1 and Std1 and also by Mig1 and Mig2 in high glucose (Fig 2E and Fig D in S1 Text).
3. HXT5 is weakly regulated by the Snf3-Rgt2 subsystem if at all [44] because all the deletions leave Hxt5’s behaviour unchanged (Fig 2F and Fig E in S1 Text). HXT5 is unlikely to be redundantly repressed by Mth1 and Std1 because deleting STD1 at glucose concentrations where Mth1 is expected to be inactive has no phenotype (Fig 2F).
4. HXT2, HXT6, and HXT7 are repressed predominately by Mth1 in low glucose (Fig 2D and 2G) and by Mig1 and Mig2 in high glucose (Figs B, F, and G in S1 Text), where they respond to deleting RGT2 and STD1 similarly to Hxt4.

Taken together, the data support the regulation of the HXTs having a bipartite structure. The high affinity transporters, HXT6 and HXT7, and the medium affinity transporters, HXT2 and HXT4, constitute one output of the system. Through Mth1 and Mig1 and Mig2, they are predominately regulated by both the high affinity sensor Snf3 and the kinase complex SNF1 and express in lower and intermediate concentrations of glucose. The low affinity transporters, HXT1 and HXT3, constitute the second output of the system. Through Std1, they are predominately regulated by the low affinity sensor Rgt2 and express at higher concentration of glucose.

## Discussion

Plate readers are a convenient, medium throughput means to measure microbial growth and gene expression, but their application is limited by the difficulties of analysing the resulting data. Here we have presented omniplate, which automates correcting OD to be proportional to the number of cells, correcting for the effects of the media, correcting fluorescence for autofluorescence, normalising per cell, and estimating growth rates as functions of time. Further, omniplate estimates errors, using the numbers of measurements and their variation, and allows meta-analysis and averaging across multiple plates, with powerful, established data structures.

As illustrations, we characterised the Monod curve for budding yeast in raffinose, demonstrating that raffinose is a convenient carbon source for controlling growth rates, and, through using fluorescent reporters to study yeast’s glucose transport, showed that the time-series data produced by plate readers can reveal aspects of gene regulation that are challenging to infer from snap-shot data.

## Availability and future directions

The software is in Python and either complements or extends that available in R [45], which focuses on calibrating and comparing with data from flow cytometers, and Matlab [46], which focuses on estimating the activities of promoters.

It is available from Python’s standard repository, PyPI, at https://pypi.org/project/omniplate as well as from gitlab. Instructions for installation, a tutorial, and documentation are at https://swainlab.bio.ed.ac.uk/software/omniplate.

## Supporting information

S1 Text Media, strains, and plots for all GFP-tagged HXTs. (PDF)
