# Supplementary material for: Analysing and meta-analysing time-series data of microbial growth and gene expression from plate readers
Source: PLoS Comput Biol. 2022 May 26;18(5):e1010138. doi: 10.1371/journal.pcbi.1010138 (PMC9176753; doi:10.1371/journal.pcbi.1010138)
Supplement: S1 Text — (PDF) [file pcbi.1010138.s001.pdf]

Text S1: Analysing and meta-analysing time-series data of microbial growth and gene expression from plate readers

Luis Fernando Montaño-Gutierrez[1], Nahuel Manzanaro Moreno[1], Iseabail L. Farquhar[1], Yu Huo[1], Lucia Bandiera[2], and Peter S. Swain[1,3]

[1]School of Biological Sciences, University of Edinburgh, United Kingdom
[2]School of Engineering, University of Edinburgh, United Kingdom
[3]Corresponding author

Plate-reader experiments

Media

Abbreviation	Composition	Use
SC	0.2% Yeast Nitrogen Base (YNB) , 0.5% ammonium sulfate	Pre-cultures
LFSC	YNB w/o riboflavin, folic acid 0.5% ammonium salts	Plate reader
XY Glucose	YEP + 0.1% adenine + 0.2% tryptophan +2% glucose	Yeast transformation

Table A. Growth media

Strain ID	in–text description	Genotype/background
SL78	BY4742 (WT)	MATα, his3Δ1, leu2Δ0, ura3Δ0, met15Δ0
SL229	BY4741 (WT)	MATa, his3Δ1, leu2Δ0, ura3Δ0, met15Δ0
SL567	SGA query strain Y6547	Matα can1Δ::pMFA1-LEU2 lyp1Δ ura3Δ0 leu2Δ0 his3Δ1 met15Δ0
SL621	rgt2Δ	SL567 rgt2::Hph
SL612	std1Δ	SL567 std1::Hph
SL618	mth1Δ	SL567 mth1::Hph
SL668	mig1Δ	SL567 mig1::Hph
SL620	mig2Δ	SL567 mig2::Hph
SL614	snf3Δ	SL567 snf3::Hph
SL498	Hxt1-GFP	SL229 HXT1-yEGFP::HIS
SL480	Hxt2-GFP	SL229 HXT2-yEGFP::HIS
SL485	Hxt3-GFP	SL229 HXT3-yEGFP::HIS
SL409	Hxt4-GFP	SL229 HXT4-yEGFP::HIS
SL487	Hxt5-GFP	SL229 HXT5-yEGFP::HIS
SL488	Hxt6-GFP	SL229 HXT6-yEGFP::HIS
SL566	Hxt7-GFP	SL229 HXT7-yEGFP::HIS
SL957	Hxt1-GFP rgt2Δ	SL498 x SL621
SL959	Hxt1-GFP std1Δ	SL498 x SL612
SL956	Hxt1-GFP mth1Δ	SL498 x SL618
SL798	Hxt1-GFP snf3Δ	SL498 x SL614
SL961	Hxt2-GFP rgt2Δ	SL480 x SL621
SL963	Hxt2-GFP std1Δ	SL480 x SL612
SL960	Hxt2-GFP mth1Δ	SL480 x SL618
SL962	Hxt2-GFP snf3Δ	SL480 x SL614
SL977	Hxt3-GFP rgt2Δ	SL485 x SL621
SL979	Hxt3-GFP std1Δ	SL485 x SL612
SL976	Hxt3-GFP mth1Δ	SL485 x SL618
SL978	Hxt3-GFP snf3Δ	SL485 x SL614
SL748	Hxt4-GFP rgt2Δ	SL409 x SL621
SL749	Hxt4-GFP std1Δ	SL409 x SL612
SL796	Hxt4-GFP mth1Δ	SL409 x SL618
SL798	Hxt4-GFP snf3Δ	SL409 x SL614
SL965	Hxt5-GFP rgt2Δ	SL487 x SL621
SL967	Hxt5-GFP std1Δ	SL487 x SL612
SL964	Hxt5-GFP mth1Δ	SL487 x SL618
SL966	Hxt5-GFP snf3Δ	SL487 x SL614
SL969	Hxt6-GFP rgt2Δ	SL488 x SL621
SL971	Hxt6-GFP std1Δ	SL488 x SL612
SL968	Hxt6-GFP mth1Δ	SL488 x SL618
SL970	Hxt6-GFP snf3Δ	SL488 x SL614
SL973	Hxt7-GFP rgt2Δ	SL566 x SL621
SL975	Hxt7-GFP std1Δ	SL566 x SL612
SL972	Hxt7-GFP mth1Δ	SL566 x SL618
SL974	Hxt7-GFP snf3Δ	SL566 x SL614

Table B. Strains used. All strains were derived from BY4741 using a synthetic genetic array (SGA) library of GFP-tagged and deletion strains [1]. We planned our markers to maximise compatibility between HIS markers with a TEF promoter and terminator (plasmid pKT128) and a hph marker driven by a ADH promoter and terminator (plasmid pYM40). Promoter and primer sequences from the *Saccharomyces* reference genome version R64-2-1 for strain S288C were obtained through the Saccharomyces Genome Database [2].

Results for each HXT-GFP strain

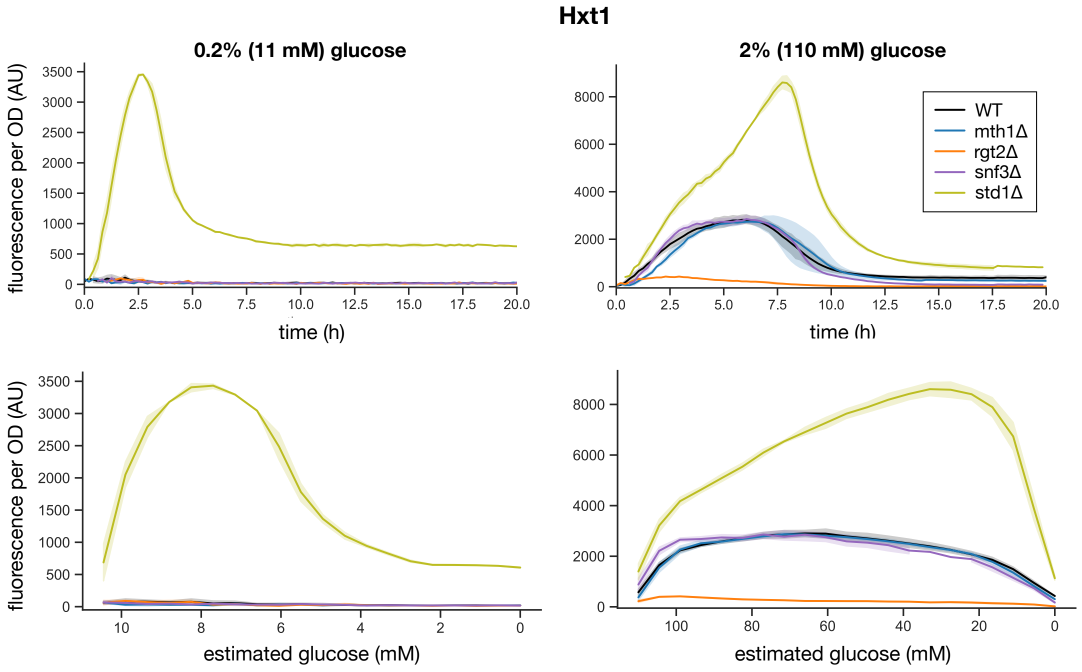

Figure A. Levels of HXT1-GFP as a function of both time and the estimated glucose concentration measured in bulk using a plate reader. Eq. 3 is used to estimate the glucose concentration from the OD and the initial concentration. Data are the mean of at least two experiments with at least three wells per experiment. Shading is the 95% confidence interval calculated by bootstrapping using the `seaborn` Python module.

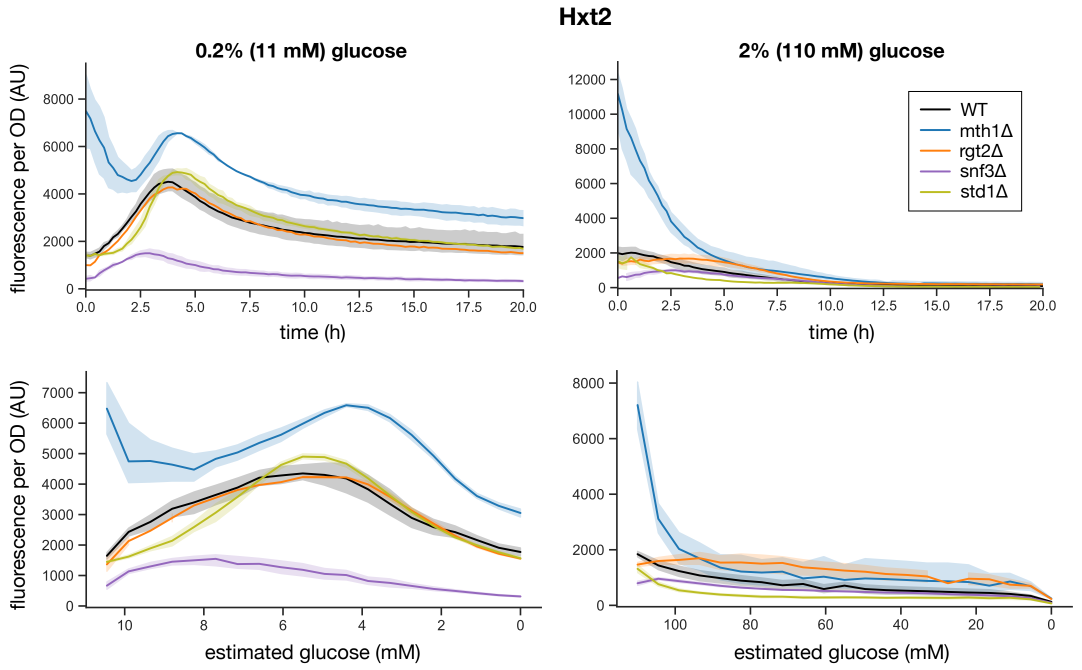

Figure B. Levels of HXT2-GFP as a function of both time and the estimated glucose concentration measured in bulk using a plate reader. Eq. 3 is used to estimate the glucose concentration from the OD and the initial concentration.

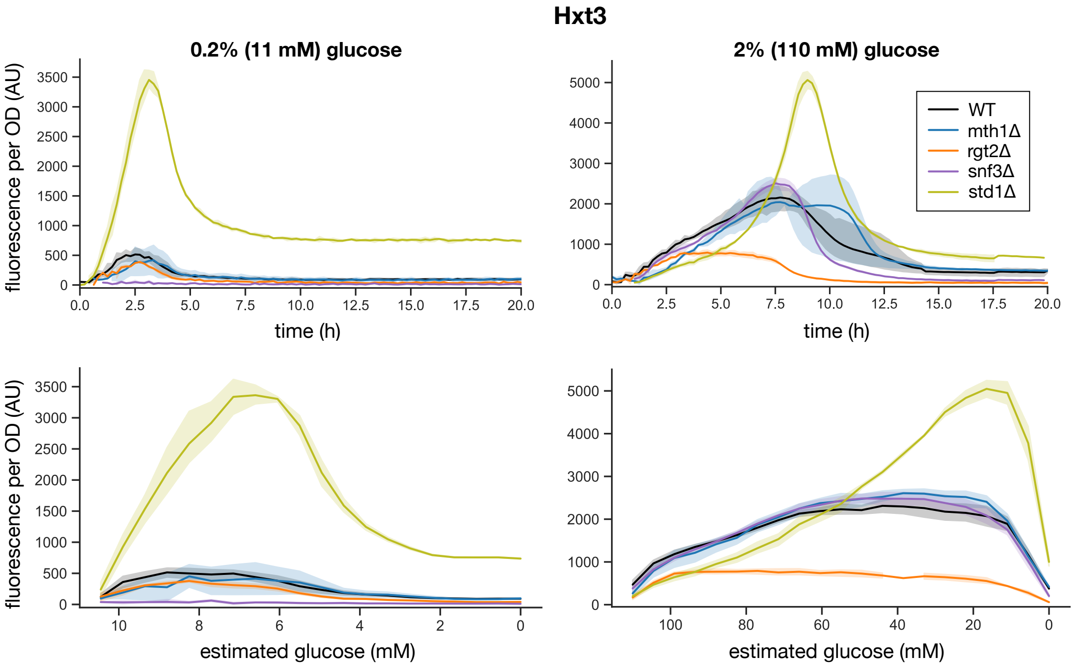

Figure C. Levels of HXT3-GFP as a function of both time and the estimated glucose concentration measured in bulk using a plate reader. Eq. 3 is used to estimate the glucose concentration from the OD and the initial concentration.

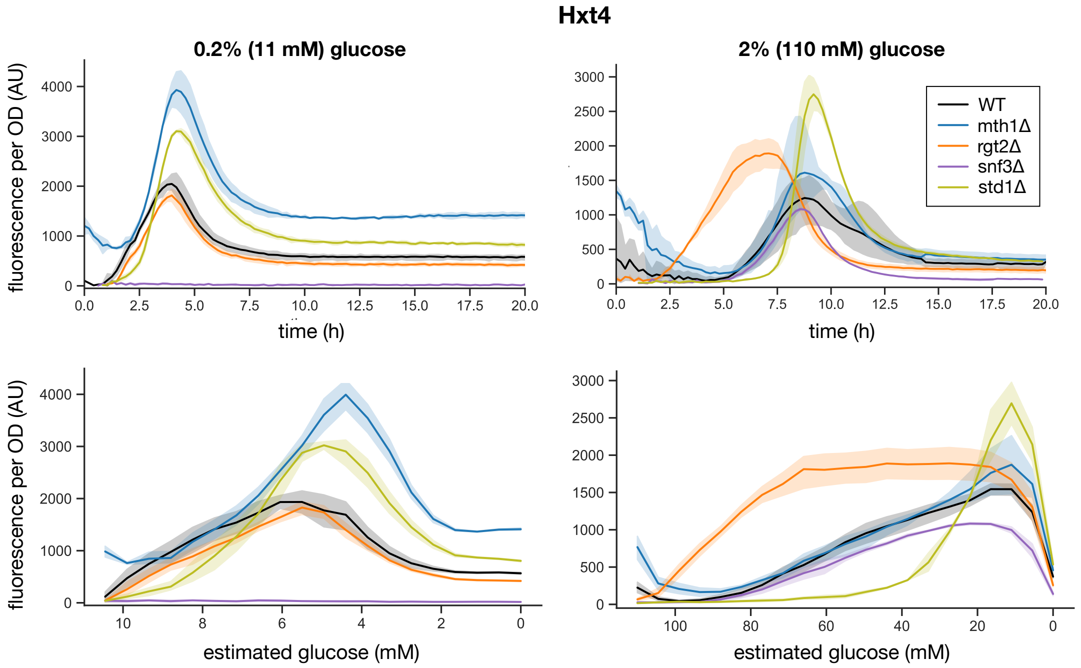

Figure D. Levels of HXT4-GFP as a function of both time and the estimated glucose concentration measured in bulk using a plate reader. Eq. 3 is used to estimate the glucose concentration from the OD and the initial concentration.

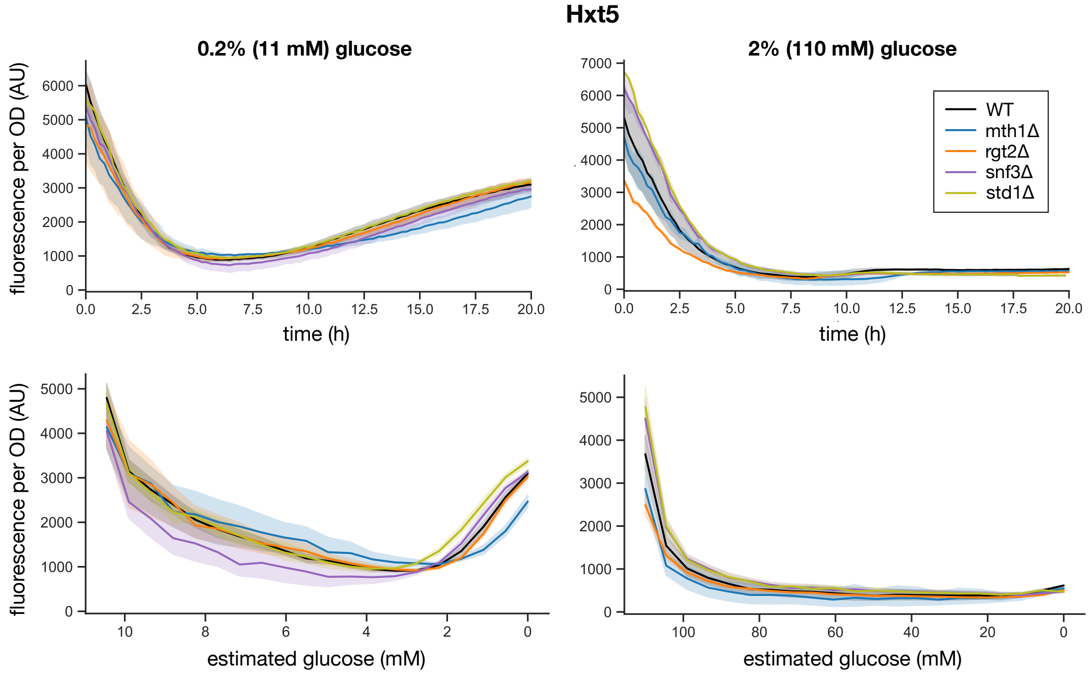

Figure E. Levels of HXT5-GFP as a function of both time and the estimated glucose concentration measured in bulk using a plate reader. Eq. 3 is used to estimate the glucose concentration from the OD and the initial concentration.

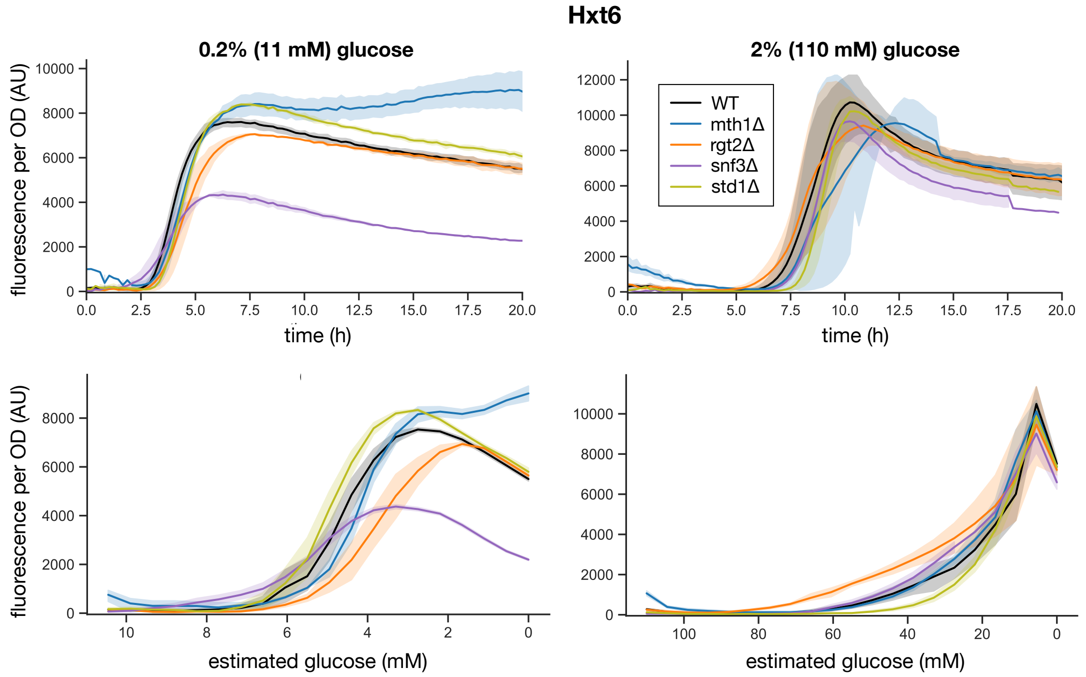

Figure F. Levels of HXT6-GFP as a function of both time and the estimated glucose concentration measured in bulk using a plate reader. Eq. 3 is used to estimate the glucose concentration from the OD and the initial concentration.

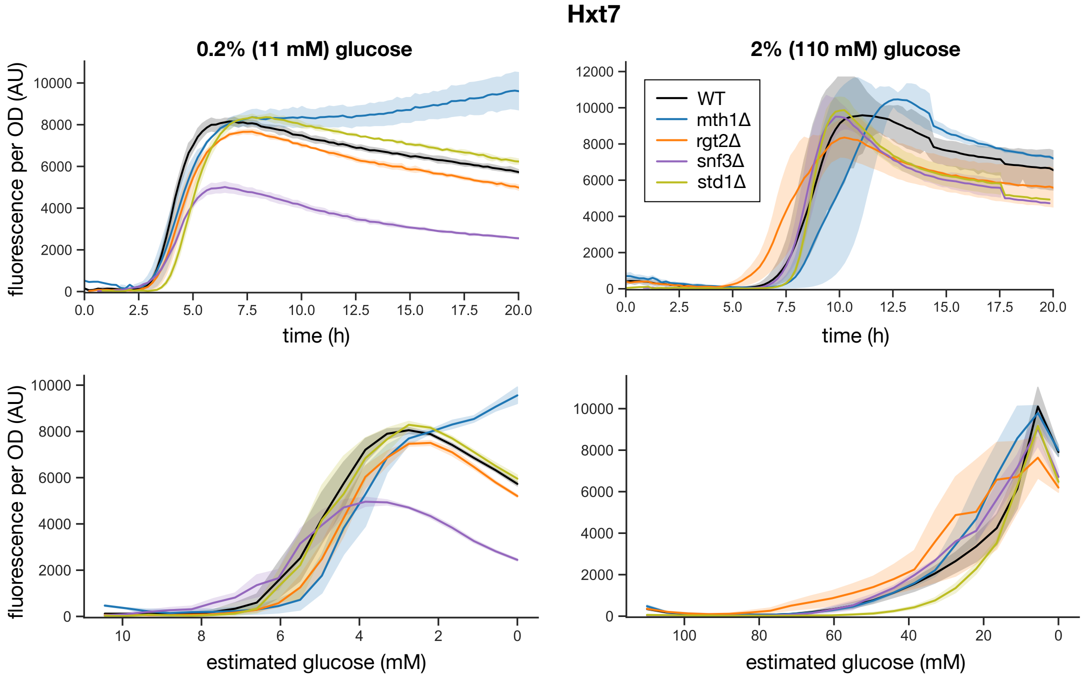

Figure G. Levels of HXT7-GFP as a function of both time and the estimated glucose concentration measured in bulk using a plate reader. Eq. 3 is used to estimate the glucose concentration from the OD and the initial concentration.

References

[1] Tong AHY, Boone C. High-throughput strain construction and systematic synthetic lethal screening in Saccharomyces cerevisiae. Met Microbiol. 2007;36:369–707.

[2] Engel SR, Dietrich FS, Fisk DG, Binkley G, Balakrishnan R, Costanzo MC, et al. The reference genome sequence of Saccharomyces cerevisiae: then and now. G3–Genes Genom Genet. 2014;4(3):389–398.
